# Predicting affective appraisals from facial expressions and physiology using machine learning

**DOI:** 10.3758/s13428-020-01435-y

**Published:** 2020-08-05

**Authors:** Laura S. F. Israel, Felix D. Schönbrodt

**Affiliations:** Department of Psychology, Ludwig-Maximilians-Universität München, Bayern, Germany

**Keywords:** Appraisal theory, Component process model, Physiology, Machine learning, Predictive modeling

## Abstract

The present study explored the interrelations between a broad set of appraisal ratings and five physiological signals, including facial EMG, electrodermal activity, and heart rate variability, that were assessed in 157 participants watching 10 emotionally charged videos. A total of 134 features were extracted from the physiological data, and a benchmark comparing different kinds of machine learning algorithms was conducted to test how well the appraisal dimensions can be predicted from these features. For 13 out of 21 appraisals, a robust positive *R*^2^ was attained, indicating that the dimensions are actually related to the considered physiological channels. The highest *R*^2^ (.407) was reached for the appraisal dimension *intrinsic pleasantness*. Moreover, the comparison of linear and nonlinear algorithms and the inspection of the links between the appraisals and single physiological features using *accumulated local effects* plots indicates that the relationship between physiology and appraisals is nonlinear. By constructing different importance measures for the assessed physiological channels, we showed that for the 13 predictable appraisals, the five channels explained different amounts of variance and that only a few blocks incrementally explained variance beyond the other physiological channels.

The cognitivist revolution during the 1960s, an intellectual movement replacing behaviorism that had dominated psychology in the first half of the twentieth century, also led to new developments in affective science (Scarantino & de Sousa, 2018). Led by Arnold (1960) and Lazarus (1966), the emotion formation process, neglected in earlier behavioristic approaches to emotions, came to the fore of research and formed the basis for the new tradition of appraisal theories. These conceive emotions as an evaluative process in which the meaning of a stimulus to the individual is determined—the relevance of a stimulus for one’s well-being is appraised in respect to personal values, needs, attachments, and goals (Moors, Ellsworth, Scherer, & Frijda, 2013). In contrast to other conceptualizations of the emotion process (e.g., Schachter & Singer, 1962), appraisal theorists place this cognitive component at the beginning of an emotional episode, resulting in bodily, motor, and motivational changes and potentially in the subjective perception of a feeling (Moors, 2009). An emotion is hence understood as a multi-componential process, integrating the cognitive appraisal with its subsequent constituents. To understand the complex emergence of emotions, much research has been conducted to learn how these components interact with each other. The main focus has been to understand how specific appraisal patterns map onto the subjective perception of emotions. Prototypical appraisal patterns for different emotion classes have been derived from both theoretical assumptions (e.g., Frijda, 1986; Roseman, 1984; Scherer, 2001; Smith & Ellsworth, 1985) and empirical data (e.g., Israel & Schönbrodt, 2019; Meuleman & Scherer, 2013). Another important objective is to examine the link between cognition and bodily changes, showing how different appraisal outcomes lead to changes in the motor system or the autonomic nervous system (ANS).

Furthering our knowledge on the connection between cognition and the body in affective states is not only fundamental to understanding emotions as a whole, but could also help in developing better tools to measure the cognitive appraisal process. To the present day, the majority of research on this topic has to rely on the use of questionnaires (e.g., Meuleman & Scherer, 2013; Scherer, 1993b, 1997; Scherer & Meuleman, 2013). Using this type of offline assessment, only constant appraisal ratings can be obtained that cannot depict potential changes in appraisal during an emotional situation. Further, the appraisal process is always evaluated in retrospect, often with a large temporal distance to the event of interest (e.g., Geneva Appraisal Questionnaire by Geneva Emotion Research Group, 2002), which potentially affects the reliability of the ratings. This demonstrates the need for the development of more indirect continuous measurement tools in the future. Before this can be achieved, though, we need to gain more insight into the relationship of bodily changes and self-reported appraisals—investigating which physical changes are predictive for which appraisal dimensions.

## The link between appraisals and physiology

The *Component Process Model* (CPM) by Scherer (1984, 2001, 2009), one of the best-known realizations of the appraisal theory, differentiates between five emotion components: the cognitive *appraisal component* that regulates the appraisal process, a *motivation component* that initiates action tendencies, a *feeling component* that comprises the subjective perception of an emotion, and two bodily components—the *physiological component* connected to efferent effects in the autonomous nervous system, and the *expression component* controlling motor expressions such as gestures, mimic, and voice. When investigating the relationship between cognition and bodily changes within the appraisal framework, the relation between the appraisal component and the two bodily components has to be considered. Therefore, the present study investigates several physiological measures, including facial *electromyography* (EMG), *electrodermal activity* (EDA), and *heart rate variability* (HRV). The latter two can clearly be associated with the physiological component of the model, while facial EMG is also used as an indicator for facial expression and can hence be associated with the expression component as well. However, as all three measures assess physiological responses, and as we are interested in the overall relationship between appraisals and bodily changes, we will hereinafter refer to them as physiological measures without this differentiation. It should be kept in mind, however, that the CPM theoretically assigns facial expressions to a separate bodily component.

Scherer (2001, 2009) assumes 16 different appraisal dimensions. For 10 of these dimensions, Scherer (2009) makes elaborate predictions on how they relate to response patterns in the physiological and the expression component. The CPM predicts, for example, that in the evaluation of the intrinsic pleasantness of a stimulus, a higher pleasantness leads to physiological changes such as heart rate deceleration, pupillary dilatation, and parted lips with pulled up corners, while an unpleasant stimulus should result in an opposite reaction with a heart rate acceleration, pupillary constriction, and lip corner depression (Scherer, 2009). As these theoretical predictions are rather speculative, different studies have tried to investigate these theoretical links in experimental settings. Van Reekum et al. (2004) induced pleasant and unpleasant as well as goal-conducive and goal-constructive events in a computer game while measuring several physiological reactions during the game. A higher skin conductance response for pleasant compared to unpleasant events was found, and obstructive events led to higher skin conductance, a stronger increase in heart rate variability, and higher pulse transit times compared to conducive events. Aue and Scherer (2008) varied the same two appraisal dimensions in a performance task in which pleasant and unpleasant pictures were presented. During the task, pictures would increase or decrease in size, where an increase in a pleasant stimulus was considered goal-conducive and a decrease in the same picture as goal-obstructive (the converse logic was applied to unpleasant pictures). The authors reported an increase in heart rate and higher activity of the zygomaticus major muscle for pleasant pictures, and higher corrugator muscle activity for unpleasant pictures. Higher zygomaticus response, higher heart rate, and higher skin conductance were found for the conducive conditions, and higher corrugator activity for the obstructive ones. Similar studies that induced appraisal outcomes in an experimental setting have been conducted by Aue, Flykt, and Scherer (2007), Delplanque et al. (2009), Gentsch, Grandjean, and Scherer (2013), Kreibig, Gendolla, and Scherer (2012), and Lanctôt and Hess (2007), as well as by Scherer, Dieckmann, Unfried, Ellgring, and Mortillaro (2019), who used encodings of facial expressions from video recordings instead of muscle activity.

Even though studies like these provide important insights into the relationship between appraisal and physiology, very few appraisals were able to be tested at a time. As the majority of these studies also used very small sample sizes, the reliability of their results can be questioned. Moreover, there was little control over whether the experimental conditions actually induced the respective appraisal, as a specific stimulus might not be pleasant, relevant, or goal-conducive to all participants, depending on their personal context. Another important downside of the experimental induction of appraisals is that not all dimensions can be analyzed, as some appraisals, such as compatibility with self-image and internal norms (an appraisal that has been proposed within Scherer’s, 2009 CPM), can hardly be induced in an experimental setting.

A different approach for examining the relations between the appraisal component and the physiological/expression component was presented by Meuleman, Moors, Fontaine, Renaud, and Scherer (2019). The authors reanalyzed a large cross-cultural data set collected with the so-called GRID tool by Fontaine, Scherer, and Soriano (2013). The data contain ratings of 24 culturally shared emotion words and their semantic meaning with regard to features of the five emotion components proposed by the CPM. Meuleman et al. (2019) assessed seven appraisal, three physiology, and five expression factors from the data set and were able to demonstrate that the physiology and expression factors were predictable, to varying degrees, from the attained appraisal factors. They also reported the type of relation for selected dimensions, such as a positive relation between the suddenness appraisal factor and the jaw drop factor. The study demonstrates the advantages of observational designs that allow for the simultaneous assessment of larger sets of appraisal dimensions, in contrast to the previously discussed empirical studies. However, it must be considered that the study does not use any actual physiological measures. The transferability of the results is therefore unclear.

Altogether, there are rather incomplete theoretical assumptions and a lack of empirical evidence on the relations between appraisal and physiology. For many appraisal dimensions, we have no predictions at all about their relation to bodily responses (either from theory or from empirical studies). In fields of research where a strong theoretical background is missing, exploratory methods can be very useful for generating new knowledge and filling in the gaps.

## Exploring the physiology–appraisal link

The goal of the present study is to take a more holistic approach to investigate the interrelations between a whole set of appraisals and measured physiological reactions by applying exploratory and data-driven methods based on machine learning on a larger sample. Machine learning modeling with features extracted from physiological data has gained popularity not only in the field of medical diagnostics (Magoulas & Prentza, 2001) but has also been applied in emotion recognition (for an overview, see Jerritta, Murugappan, Nagarajan, & Wan, 2011). Studies focusing on the latter induce emotional states using auditory, visual, or audiovisual material during which different physiological signals are assessed, and participants can name their perceived emotional state afterward. Subsequently, different features characterizing the signals are extracted from the data and used to predict the emotional output using different machine learning algorithms. The evaluation of these models can then tell how well emotion categories can be predicted from this kind of data and validate the assumed link between the perceived feeling and bodily responses during an emotional situation. Furthermore, it can be assessed which features are most important in predicting an emotion category.

To establish the link between physiological responses and appraisal, the same approach can be applied. For this purpose, we presented emotionally charged video material to participants while measuring HRV, EDA, and EMG on three facial sites—the zygomaticus major site, the corrugator supercilii site, and the frontalis muscle site. All five channels have been identified as affect-related and have been used previously in the prediction of emotions (e.g., Haag, Goronzy, Schaich, & Williams, 2004; Kim & Andre, 2008; Rigas, Katsis, Ganiatsas, & Fotiadis, 2007). The three measured EMG sites are physiologically connected to the motions of smiling (zygomaticus major), frowning (corrugator supercilii), the raising of eyebrows, indicating expressions of surprise (frontalis; Murata, Saito, Schug, Ogawa, & Kameda, 2016), and many other facial expressions. They are known to enable the identification of the valence of a stimulus and the detection of mental stress (Egger, Ley, & Hanke, 2019). The CPM marks several facial responses as outcomes of specific appraisals (for a detailed description, see Table 1 in Scherer & Ellgring, 2007), and the discussed empirical studies substantiate this interrelation (Aue et al., 2007; Aue & Scherer, 2008; van Reekum et al., 2004). EDA, the measure of skin conductivity, is also known to be related to affective reactions, especially eccrine glands measured on the palms that decrease during relaxation and increase during phases of exertion (Egger et al., 2019). A link between EDA and different appraisals such as conduciveness, goal relevance, novelty, and pleasantness of stimuli has been reported in several empirical studies as well (Aue & Scherer, 2008; Scherer, 2009; van Reekum et al., 2004). As changes in heartbeat are modulated by the sympathetic and parasympathetic system (Rainville, Bechara, Naqvi, & Damasio, 2006), HRV, which measures changes in beat-to-beat intervals, has been used effectively for the detection of emotional arousal (Egger et al., 2019). Several theoretical relations between ECG features and appraisals have been predicted by the CPM, also implying a connection between the cognitive evaluation of a stimulus and heart rate (Scherer, 2009). Consequently, all physiological measures collected in the present study are closely interlinked with affect and are presumably predictive for different appraisal outcomes.

After the measurement of the physiological responses to each video, we assessed 15 different appraisal dimensions that have been proposed by the CPM: *suddenness* (How sudden does an event occur?), *familiarity* (How familiar is the event?), *predictability* (How predictable was the occurrence of an event?), *intrinsic pleasantness* (How pleasant was an event?), *goal/need importance* (How relevant is an event for the achievement of current goals?), *cause agent* (Who or what caused an event?), *cause motive* (Was an event caused intentionally?), *outcome probability* (Can potential consequences of an event be determined?)*, discrepancy from expectation* (Did an event contradict previously built expectations?), *conduciveness* (Does an event help to attain personal goals?)*, urgency* (Is it urgent to react to an event?), *control* (Can the outcomes of an event be controlled?)*, adjustment* (Is it possible to adjust to the outcomes of an event?), *compatibility with external* and *internal standards* (Is an event compatible with social norms and laws or self-image?). See Scherer (2001) for a more thorough description of the appraisals. For the assessment of these appraisal dimensions, a modified version of the *Geneva Appraisal Questionnaire* (GAQ; Geneva Emotion Research Group, 2002) was used. We extracted 134 features from the five assessed physiological channels and predicted each appraisal dimension using both a tree-based and a linear machine learning model, reporting the overall cross-validated model performance for each dimension. If a link between the measured physiological signals and an appraisal dimension exists, an adequate model should be able to predict the appraisal outcome to some degree. The observational design of the study does not allow us to investigate the causal direction between appraisal and physiological features. For this reason, and because we use a large number of features for each physiological channel^1^, we modeled the appraisal–physiology link in the reverse direction (i.e., physiology predicts appraisal, although theory mainly proposes the reverse causal direction). We also constructed two different importance measures depicting the significance of each of the five physiological channels in the appraisal predictions and exemplarily analyzed the type of relationship between the appraisal dimensions and selected features.

With this data-driven approach, we are, in contrast to earlier studies, able to investigate a whole set of appraisals at once and also do not rely on uncertain appraisal inductions. We are able to analyze the appraisal–physiology link for several dimensions that have not yet been tested empirically—many of which cannot be tested in a classical experimental design. In addition, we consider not only nonlinear relations in our data but can also account for complex interactions. Moreover, as all performance and importance measures are computed on out-of-sample data, our results and the derived conclusions can be considered as more robust against overfitting and therefore as more generalizable. With the exploratory analysis of the appraisal–physiology link, we hope to generate new knowledge in a rather fragmented section of emotion research.

## Method

Reproducible scripts, open data, and open materials (including codebooks and video stimuli) are provided via our OSF repository at https://osf.io/pbt9r/.

### Participants

A total of 172 participants were recruited for the present study that either received a payment or a participation certificate. The sample size was based on available funding. As each participant viewed and rated 10 videos, 1720 observations resulted from this data collection. Due to technical problems such as signal interruption or corrupted files that caused one or more of the physiological signals to be missing (EMG, EDA, or RR data), several observations and participants had to be excluded. The final sample consisted of 157 participants (female = 95) and 1556 observations. The majority of subjects (94%) were students at the Ludwig-Maximilians-Universität München (32% of whom were psychology students), with an average age of 25.47 years (range = 19–62).

### Stimulus material

To produce different appraisal outcomes and physiological reactions, emotional video sequences were used to induce various emotional states. Videos marked with a Creative Commons CC-BY license, which allows modification and redistribution of the content, were gathered during an extensive online web search on the video-sharing service YouTube (YouTube, n.d.). To create variance in the video content, videos were broadly chosen by their potential emotional effect on the viewer—fitting to the four emotion terms fear, sadness, disgust, and joy. To control for culture and language effects, only German or language-free videos were included. Video sequences were cut to not exceed a maximum length of 30 s. In an online study, a selection of 20 videos was pretested. The videos were presented in randomized order to 28 participants (female = 17), who were asked to rate the intensity of their emotional experience during the observation and answer a questionnaire constructed to assess the 16 appraisal dimensions implied by the CPM (see Procedure section for a detailed description of the questionnaire). Participants were also asked to label the videos with an emotion term—these emotion labels were, however, not considered in the further video selection. In total, 211 video ratings were collected in the pretest, with 7–15 ratings per video. To predict the appraisal dimensions from the physiological data, the ratings of each appraisal had to show a sufficient amount of variance. In addition, the video content had to be intensive enough to elicit a measurable physiological reaction. Based on these two criteria, a set of eight videos was selected, showing both high variance in the appraisal ratings and high affective intensity. Even though all positive videos were rated as less intense and showed lower appraisal variance, two positive videos were also included to balance out the valence of the data set. Overall, 10 emotional videos with a mean length of 24.8 s (range = 10.5–30.5) were included. All videos are provided in our OSF repository at https://osf.io/pbt9r/.

### Apparatus

For the measurement of the EMG and EDA signals, pre-gelled disposable electrodes with a .8 cm Ag/AgCl detection surface were used. For common-mode rejection, all sites were measured using a bipolar recording scheme. EMG electrode placement for corrugator, frontalis, zygomaticus, and ground electrode was conducted following the guidelines by Fridlund and Cacioppo (1986). Electrodes for the bipolar skin conductance measurement were placed on the thenar and hypothenar eminences of the non-dominant hand of the participants (Fowles et al., 1981). A fixture on the non-dominant hand was conducted to prevent any interference with the electrodermal measurement during the tasks. The skin was prepared by cleaning the measurement sites with alcohol wipes (70% isopropanol) and applying an abrasive electrode gel to lower the skin impedance.

For data collection, a Biopac BioNomadix MP160 data acquisition system with two wireless two-channel EMG transmitters and one wireless PPG and EDA transmitter was used (Kremer, Mullins, Macy, Findlay, & Peterlin, 2019). Channel calibration and data acquisition were conducted using the corresponding AcqKnowledge software (version 5.0.2; Kremer et al., 2019). In accordance with the Nyquist theorem, which indicates that a sinusoid signal should be sampled at least at twice its frequency for correct reconstruction, signals were sampled at a frequency of 1000 Hz (De Luca, 2003). For the HRV measurement, a Polar H10 heart rate sensor and a Polar V800 heart rate monitor were used, which have been proven to be consistent with measures derived from an ECG system (Giles, Draper, & Neil, 2016). The experimental program to present the videos and assess the subsequent rating of the appraisal dimensions was implemented using E-Prime 2.0 software (Schneider, Eschman, & Zuccolotto, 2012). To synchronize the physiological data collected with AcqKnowledge and the videos presented in E-Prime, Observer XT (version 14.1.1121; Zimmerman, Bolhuis, Willemsen, Meyer, & Noldus, 2009), a software for behavioral coding and event logging, was used to control and integrate the two data streams. The preliminary questionnaire sent to the participants was provided via the FormR survey framework (Arslan, Tata, & Walther, 2018).

### Procedure

Each participant received a randomized code consisting of four numerals to use as identification throughout the two-part study. First, participants completed an online questionnaire from home. In this preliminary survey, subjects were informed about the study and gave their consent to participate and to the publication of their fully anonymized data. Subsequently, all relevant demographic information and further variables not included in the present study (e.g., personality, motives, emotional sensitivity^2^) were collected. For the second part of the study, each participant was invited to a laboratory. After receiving a brief introduction, the subject was asked to put on the Polar strap with the heart rate sensor. The investigator then prepared the subject’s skin, applied the electrodes as described, and affixed the two EMG transmitters to the head and the EDA transmitter to the wrist of the non-dominant hand of the participant.

Before the start of testing, a calibration of the EMG and EDA transmitters was conducted, during which the transmitter leads were connected to the electrodes. Participants were instructed to perform different facial movements to test whether contractions would result in peaks in the respective signals. During this test phase, the investigator avoided using any emotion-related terms such as *smiling* or *frowning*, in order to bias the subject as little as possible. If a reliable signal was detected, the participant was seated in front of a computer screen and the heart rate measurement and the experimental program was started. To prevent subjects from feeling that they were being observed, the investigator monitored the physiological signal from a separate area during the following testing, intervening only if noise occurred or when electrodes needed to be reattached. Subjects were advised to place their non-dominant hand with the EDA transmitter on the table and move this hand as little as possible, answering and navigating through the study using their dominant hand on a keyboard in front of them. The participants followed a standardized instruction provided to them on screen, starting with a baseline measurement of two minutes, in which participants were instructed to close their eyes and relax. Afterward, the 10 videos were presented in randomized order, each followed by a questionnaire for the assessment of the appraisal dimensions. In addition, subjects were asked to label the emotion they felt during the video and answer items relating to their immersion during the viewing of the video—these ratings had no relevance to the present study.

The presented appraisal questionnaire was based on the German version of the GAQ (Geneva Emotion Research Group, 2002). The GAQ was developed to assess through recall and verbal report as much information as possible about the appraisal process during an emotional episode. The original questionnaire, consisting of 26 items, asks respondents to recall an arbitrary moment in the past when an intense emotion was experienced and to rate the respective experience on the 16 appraisal dimensions of the CPM (e.g., *At the time of experiencing the emotion, did you think that the event happened very suddenly and abruptly?).* For the purpose of the present study, one item for each of the appraisal dimensions was selected from the questionnaire and altered slightly to fit the video rating context (e.g., *Did you think that the events in the video happened very suddenly and abruptly?*). Only the dimension *cause agent*, which identifies who the agent of an evaluated event is, was assessed using three different items, identifying whether the protagonist of a video, a person different from the protagonist, or natural forces caused the events. Furthermore, we constructed an additional item for each of the four dimensions *goal/need importance*, *conduciveness*, *urgency*, and *adjustment*, that asked the participant to rate the respective dimension from the perspective of the protagonist in the video (e.g., *Can you live with, and adjust to, the consequences of the displayed events? Do you think that the protagonist can live with, and adjust to, the consequences of the events?*). As the participant’s goals and actions were probably not strongly affected by the passive viewing of the mostly fictional video content, we suspected that for these dimensions, the assumed effect on the protagonist (e.g., the potential outcome of the event with regard to the character) might be more relevant to the emotional evaluation of the video then the evaluation of the effect on the participants themselves—especially if the viewer felt strongly involved. The dimension *power*, which evaluates the degree to which the rater can influence a situation himself, was excluded from the questionnaire. All items were rated on a five-point scale ranging from *not at all*, *moderately* to *extremely*. In addition, participants were able to indicate that a question did not apply to the content of the video. Participants were also asked to indicate whether they experienced an emotion during the viewing of the video and to rate the intensity of their emotional experience on a five-point scale (if an emotion was present).

All items of the appraisal questionnaire (both the original German ones and their English translation) and the respective appraisal dimensions can be found in the codebook of our data set in our electronic appendix.

### Data preprocessing

The preprocessing and all further analyses were conducted in R (version 3.4.2; R Core Team, 2018). For each participant, the physiological signals (EMG, EDA, HRV) during the viewing of each video were extracted using E-Prime timestamps, indicating the onset and offset of each video during the experiment. All data points assessed during other phases of the experiment were discarded except for the baseline measurement. To determine the noise contamination in the EMG data, frequency spectra were calculated using the *spec* function from the *seewave* package (Sueur, Aubin, & Simonis, 2008). The signals showed high noise contamination due to movement artifacts in the frequency range below 40 Hz as well as electromagnetic noise at 50 Hz. Therefore, a Butterworth high-pass filter with a cutoff frequency of 40 Hz was applied using the *highpass* function from the *biosignalEMG* package (Guerrero & Macias-Diaz, 2018). To filter out electromagnetic noise, a notch filter with a width of .5 Hz was applied at the respective frequency using the *bwfilter* function from the *seewave* package (Sueur et al., 2008). In line with the recommendations of Fridlund and Cacioppo (1986), we also applied a low-pass filter at 250 Hz using the *lowpass* function from the *biosignalEMG* package (Guerrero & Macias-Diaz, 2018). In addition, a baseline correction using the mean level of activation during the baseline measurement was applied to the EMG channels using the *dcbiasremoval* function from the *biosignalEMG* package (Guerrero & Macias-Diaz, 2018). As some residues of movement artifacts remained in the data, and because these artifacts might influence features based on the amplitude of the signal, we added two more robust amplitude features containing a 20% trimming of the signal (see next section) to the feature set. To remove the tonic level from the EDA signal, a high-pass filter at .5 Hz was applied to the data, as recommended by Braithwaite, Watson, Jones, and Rowe (2013), again using the *bwfilter* from the *seewave* package (Sueur et al., 2008).

### Physiological features

For the description of the different physiological signals, several sets of features were implemented. For the characterization of the EMG signals time and frequency domain, 32 different features were calculated (see Table 1 for an overview of all features). The specific computation of these features is based on the formulas provided by Phinyomark, Limsakul, and Phukpattaranont (2009) and Phinyomark, Phukpattaranont, and Limsakul (2012). Where necessary, features were normalized to make them independent of the length of the time series. While most of these features are used for the characterization of time series data in general, some of them are more specifically applied to EMG data. As only the percentage Wilson amplitude and the zero-crossing percentage (with the .005 mV threshold) yielded zero variance on the EDA data, however, all other features were deemed appropriate to describe the skin conductance signal as well. For the analysis of the heart rate variability data, we implemented a different set of features based on the recommendations of Vollmer (2015). Overall, 134 features were calculated—32 for each of the EMG channels, 30 for the EDA data, and 8 for the heart rate variability data. See the R scripts provided in our electronic appendix for a formal description of the feature set.

**Table 1:** Features extracted from EMG, EDA, and HRV channels

Features	EMG	EDA	HRV
Mean absolute value	X	X	
20% trimmed mean value	X	X	
Mean absolute value attenuated with a moving-window-20%-trimmed-mean filter	X	X	
Simple square integral	X	X	
Variance	X	X	
Absolute value of the 3rd – 5th spectral movement	X	X	
1st – 4th order autoregressive coefficients	X	X	
Root mean square	X	X	
Log detector	X	X	
Percentage waveform length	X	X	
Average amplitude change	X	X	
Difference absolute standard deviation value	X	X	
Percentage zero-crossings	X	X	
Percentage zero-crossings (.005 mV threshold)	X		
Percentage slope sign changes	X	X	
Myopulse percentage	X	X	
Percentage Wilson amplitude	X		
Median frequency of the amplitude spectrum	X	X	
Mean frequency of the amplitude spectrum	X	X	
Median frequency of the frequency spectrum	X	X	
Mean frequency of the frequency spectrum	X	X	
Peak frequency	X	X	
Mean power	X	X	
Total power	X	X	
1st – 3rd spectral movement	X	X	
Standard deviation of RR intervals			X
Root mean square of RR intervals			X
Percentage of successive RR intervals differing more than 50 ms			X
Ratio of the power of the low and high-frequency bands			X
Triangular interpolation of the discrete distribution of the RR intervals			X
Ratio of the standard deviation along the identity line and the standard deviation of the perpendicular axis of the Poincaré plot			X
Total number of RR intervals divided by the number of intervals in the modal bin			X
Total number of relative RR intervals divided by the number of intervals in the modal bin			X

### Machine learning modeling

#### Benchmark

Most appraisal dimensions were assessed by a single item in our questionnaire. For the dimensions assessed with more than one item, we calculated inter-item correlations. As all correlations were low (all *r* < .4), we refrained from aggregating the items and included each of them as a separate appraisal dimension (for a similar approach, see Scherer & Meuleman, 2013). All negative poled items were reversed. For each of the 21 appraisal dimensions, we constructed a regression task using the 134 physiological features as predictors. In each task, we excluded all observations with a missing rating (*does not apply* answer) in the respective appraisal dimension. Hence, the different tasks compromised data sets of different sizes that ranged from *n* = 1556 for *pleasantness* to *n* = 948 for *internal standards* (*M* = 1337.6). For each of the 21 tasks, a benchmark experiment was conducted that compared a baseline model, a featureless learner (FL) that predicted the mean, to a linear ridge regression model (RIDGE) and a random forest model (RF), able to represent complex interactions and nonlinearity, using the *mlr* package (Bischl et al., 2016). For all models, the default hyperparameter settings were used.^3^ To evaluate the performance of the models, we conducted a 20 × 5 cross-validation and report the aggregated *R*^2^. As our data set contained several observations per subject, we blocked the samples by subject within each fold to take into account the nested structure of the data.^4^ As the preprocessing of the physiological data might not be sufficient to fully eliminate artifacts in our data, and because the linear model used in the benchmark seemed to be strongly affected by outliers in the data, we added an additional preprocessing step for the RIDGE model.^5^ First, an outlier analysis was conducted on the 134 features, eliminating all values that were more than three standard deviations away from the mean of the feature. These missing values (1.2% of the data) were subsequently imputed within each fold by using random numbers drawn from the remaining empirical distribution of the feature. The RF model that reached the highest performance for all appraisal dimensions was selected for all further analyses. To determine for which appraisal dimensions the RF was able to robustly reach a positive *R*^2^ and hence was able to explain variance in the appraisals, we looked at the variation of *R*^2^ scores within the 100 cross-validation folds. To consider an appraisal as robustly predictable, we determined that at least 85% of the attained *R*^2^ values should be positive (i.e., the 15% quantile should lie above 0).

### Blocked feature importance

In a second step, we analyzed how strongly the physiological channels contributed to the prediction of the appraisal dimensions that attained a positive *R*^2^ in the previous analysis. For this purpose, we constructed two blocked permutation importance measures also based on the *R*^2^ that could quantify the impact of each of the five physiological signals (zygomaticus, corrugator, frontalis, EDA, and HRV) summarizing all features of the respective channel.

The first channel-based importance measure, $$ {R}_B^2, $$ aims to quantify how well a physiological channel can predict an appraisal dimension in general. To this end, we selected only the features calculated from the physiological channel of interest (e.g., all corrugator features) and trained the RF model on 60% of the data using only the selected feature subset. Subsequently, the *R*^2^ was assessed on the remaining 40% test sample. The performance was calculated 100 times using different random splits and subsequently averaged (in order to avoid hold-out test sets that were too small and unstable, we chose a 40% test set instead of the previously applied 20% test set):

$$ {R}_B^2=\frac{\sum_{i=1}^{100}{R}_{B,i}^2}{100} $$

where

*B*: is the block that contains all variables of the physiological channel of interest, and
$$ {R}_{B,i}^2 $$
is the out-of-sample *R*^2^ of the model trained with only the variables of *B* in the *i*th repetition.

${R}_B^2$ shows how much variance can be explained by the variable block in the absence of any other information, and hence can be considered as a kind of “main effect” of the physiological channel, representing the overall variance that can be explained by the predictors of the channels and all interactions within the feature block.

The second channel-based importance measure, $\Delta  {R}_B^2$, aims to quantify the variance that can be uniquely explained by the channel beyond all other channels. For the computation, we again randomly split the data set into a training set holding 60% of the data and a test set holding the remaining 40%. First, the RF is trained with all the available features and the out-of-sample *R*^2^ is assessed. In a second step, the out-of-sample performance of the model trained with all features that do not belong to the physiological channel of interest (e.g., all frontalis, zygomaticus, EDA, and HRV features but not the corrugator features) is assessed. To quantify the importance of the variable block of interest, the difference between the two *R*^2^ values is calculated. For a more robust assessment, the calculation is again repeated over 100 iterations and aggregated subsequently, as shown in the following formula:

$$ \Delta  {R}_B^2=\frac{\sum_{i=1}^{100}\left({R}_i^2-{R}_{\neg B,i}^2\right)}{100} $$

where

*B*: is the block that contains all variables of the physiological channel of interest,
$$ {R}_i^2 $$
is the out-of-sample *R*^2^ of the model trained with all features in the *i*th repetition, and
$$ {R}_{\neg B,i}^2 $$
is the out-of-sample *R*^2^ of the model trained without the variables of block *B* in the *i*th repetition.

As the second model is trained and validated with all features except the variable block of interest, ${R}_{\neg B,i}^2$ represents the variance that can be explained by all other variables and all their interactions. The difference in *R*^2^ between the complete model and the partial model consequently represents the variance that can be explained by the block of interest (and its interactions with other blocks) beyond all other variables. $\Delta  {R}_B^2$ hence represents the incremental variance that is uniquely explained by the physiological channel, while ${R}_B^2$ also includes the shared variance that can also be explained by other blocks. A similar importance calculation has been recommended by Yarkoni and Westfall (2017). For the calculation of both importance measures, observations were again blocked for subjects. In addition, we again applied a robustness measure by only reporting the importance of dimensions for which the attained ${R}_B^2$ or $\Delta  {R}_B^2$ were positive in at least 85% of the iterations.

#### Accumulated local effects plots

As the *R*^*2*^ feature importance only gives information about the relevance of the feature blocks but not about the direction and type of the relations between the appraisals and the physiological channel, we also report *accumulated local effects* (ALE) plots that visualize for given values of the feature the effect on the prediction of the outcome variable (i.e., appraisal dimension; Molnar, 2019). As this additional step was conducted to gain more insight into the machine learning models, we focus on features that are easy to interpret from both a mathematical and a physiological perspective. The most straightforward interpretation can be attained by looking at features describing the amplitude height (i.e., mean absolute value, simple squared integral, root mean squared signal, absolute value of the 3rd–5th spectral movement, and log detector), as these are clearly associated with muscle contraction for EMG (Day, 2002) and sympathetic activity or arousal for EDA (Benedek & Kaernbach, 2010). We also considered all time-domain HRV features, as they all describe the amount of variability in subsequent heartbeat intervals, excluding the high- and low-frequency band ratio and the nonlinear measure based on the Poincaré plot. We calculated the feature importance for each related amplitude as well as the HRV features and selected the one with the most robust importance (yielding a positive importance in at least 85 of 100 iterations) for each of the appraisals that yielded a sufficient overall performance. To this end, a feature-based importance measure similar to the ${R}_B^2$ was used, calculating the *R*^2^ for an RF model with only the feature of interest as a predictor. To prevent overfitting in these single-feature models, we restricted the tree depth of the RF to three. We report the ALE plots of the best feature within each appraisal dimension using the *iml* package (Molnar, Bischl, & Casalicchio, 2018). The plots were again calculated from the RF model with only the respective feature as a predictor and the tree depth restricted to three. To prevent extrapolation in regions of sparse data for the feature, we only plotted data within the 5% and 95% quantiles of the feature.

## Results

Descriptive statistics (mean and standard deviation) of the 21 assessed appraisal dimensions and the 10 videos, as well as the sample sizes of the appraisal subsets used in the different appraisal prediction models, are presented in Table 2. The presented mean appraisal ratings vary between the videos due to the differences in content. Moreover, a substantial between-subject variance can be observed for each appraisal and each video (SD), demonstrating that the videos were still appraised differently by the participants. In only 30 of the1556 observations in the data set, participants reported that they did not experience an emotion during the video.

**Table 2:** Mean and standard deviation for the 21 appraisals and the 10 videos as well the sample size used in the fitting of the respective model

Appraisal	Vid 1	Vid 2	Vid 3	Vid 4	Vid 5	Vid 6	Vid 7	Vid 8	Vid 9	Vid 10	*N*
	M (SD)	M (SD)	M (SD)	M (SD)	M (SD)	M (SD)	M (SD)	M (SD)	M (SD)	M (SD)	
Pleasantness	2.67 (0.97)	1.63 (1.05)	2.72 (1.04)	3.11 (1.12)	3.38 (1.50)	4.78 (0.45)	2.27 (1.01)	4.32 (1.14)	4.19 (0.88)	4.53 (0.76)	1556
Internal standards	3.15 (1.20)	3.27 (1.30)	3.34 (1.39)	3.32 (1.18)	2.58 (1.29)	4.62 (0.77)	3.53 (1.42)	4.03 (1.29)	4.07 (0.96)	4.51 (0.98)	948
Conduciveness (protagonist)	3.28 (1.10)	2.07 (1.28)	1.76 (1.17)	2.56 (1.28)	1.47 (0.85)	3.22 (1.58)	1.97 (1.28)	1.37 (0.86)	3.67 (1.37)	1.57 (1.11)	1556
External standards	3.14 (1.21)	4.67 (0.80)	4.25 (1.13)	3.10 (1.31)	4.26 (1.00)	3.14 (1.46)	4.17 (1.08)	1.59 (1.14)	1.69 (1.21)	3.26 (1.14)	1106
Cause: motive	1.79 (1.04)	1.99 (1.36)	1.83 (1.00)	1.53 (0.87)	2.83 (1.59)	3.92 (1.26)	1.88 (0.96)	4.31 (1.11)	1.34 (0.79)	3.75 (1.21)	1195
Urgency (protagonist)	2.46 (1.11)	2.28 (1.09)	4.26 (0.93)	2.75 (1.24)	4.39 (0.91)	3.34 (1.26)	4.26 (1.04)	4.17 (1.42)	1.85 (1.44)	3.80 (1.24)	1202
Cause: agent (protagonist)	2.56 (0.76)	2.71 (0.99)	2.49 (0.77)	2.63 (0.70)	2.40 (0.99)	3.16 (0.68)	2.56 (0.77)	2.84 (0.56)	3.27 (0.89)	3.52 (0.92)	1348
Adjustment (protagonist)	1.40 (0.74)	1.23 (0.61)	1.47 (0.76)	2.04 (0.97)	2.02 (0.95)	4.32 (0.77)	1.28 (0.70)	3.24 (0.96)	3.82 (1.25)	3.85 (0.95)	1254
Suddenness	4.36 (0.84)	3.97 (1.49)	3.33 (1.19)	4.34 (0.85)	4.58 (0.83)	2.83 (1.45)	2.87 (1.26)	3.89 (1.32)	3.31 (1.33)	3.81 (1.31)	1475
Conduciveness (self)	3.37 (1.03)	3.62 (1.08)	3.28 (1.08)	2.85 (1.15)	2.85 (1.27)	3.58 (1.27)	3.91 (1.07)	3.38 (1.34)	3.82 (1.06)	4.05 (1.06)	1556
Cause: agent (other person)	3.09 (1.61)	1.88 (1.27)	2.42 (1.44)	2.90 (1.40)	1.87 (1.26)	4.73 (0.79)	3.30 (1.56)	4.38 (1.15)	4.81 (0.64)	4.79 (0.57)	1357
Goal/need importance (protagonist)	2.95 (1.34)	3.46 (1.37)	3.12 (1.40)	3.20 (1.31)	2.28 (1.42)	2.72 (1.55)	3.32 (1.53)	3.20 (1.44)	3.91 (1.28)	4.16 (1.17)	1364
Familiarity	2.12 (1.04)	2.43 (1.22)	1.72 (1.07)	2.28 (1.02)	2.81 (1.22)	1.66 (0.89)	1.49 (0.88)	1.30 (0.66)	2.30 (1.12)	1.70 (1.00)	1528
Discrepancy from expectation	4.77 (0.59)	4.77 (0.77)	4.28 (0.94)	4.01 (1.21)	3.17 (1.52)	4.43 (0.96)	4.43 (0.90)	1.77 (0.96)	4.40 (0.96)	2.43 (1.26)	1448
Cause: agent (nature)	1.99 (1.22)	2.02 (1.34)	1.94 (1.10)	1.98 (1.05)	1.30 (0.76)	4.48 (0.85)	2.02 (1.09)	3.70 (1.43)	4.47 (0.80)	4.47 (1.02)	1144
Adjustment (self)	2.79 (1.12)	3.47 (1.12)	2.75 (1.13)	2.73 (1.18)	3.49 (1.14)	4.17 (0.93)	3.21 (1.26)	4.04 (1.11)	3.38 (1.19)	3.92 (1.10)	1122
Predictability	1.74 (0.70)	1.94 (0.82)	1.94 (0.87)	1.90 (0.71)	1.33 (0.60)	2.82 (1.02)	2.04 (0.97)	1.67 (0.80)	3.42 (1.15)	4.47 (0.83)	1501
Outcome probability	2.77 (1.19)	3.32 (1.27)	2.47 (1.20)	2.15 (1.12)	2.60 (1.29)	3.62 (1.22)	3.28 (1.35)	3.41 (1.26)	3.44 (1.19)	3.60 (1.28)	1398
Control	2.87 (1.30)	2.64 (1.37)	3.26 (1.20)	2.28 (1.21)	2.97 (1.33)	2.89 (1.39)	2.98 (1.30)	2.13 (1.39)	1.67 (1.03)	1.49 (0.94)	1154
Goal/need importance (self)	4.72 (0.67)	3.63 (1.57)	4.47 (0.82)	3.18 (1.35)	3.32 (1.38)	4.65 (0.67)	4.62 (0.82)	2.30 (1.35)	3.16 (1.39)	2.42 (1.31)	1492
Urgency (self)	2.15 (1.22)	2.59 (1.32)	1.68 (1.11)	2.26 (1.17)	2.40 (1.31)	1.53 (0.97)	1.53 (1.01)	1.41 (0.88)	2.22 (1.19)	1.61 (1.04)	1386

Note: Vid 1 = “Neg_617_Trim”; Vid 2 = “Neg_601_D”; Vid 3 = “Neg_113_Trim”; Vid 4 = “Neg_601”; Vid 5 = “Neg_618_Trim”; Vid 6 = “Neg_214”; Vid 7 = “Neg_121_Trim”; Vid 8 = “Neg_212_Trim”; Vid 9 = “Pos_309_D”; Vid 10 = “Pos_317_D”

**Fig. 1:**
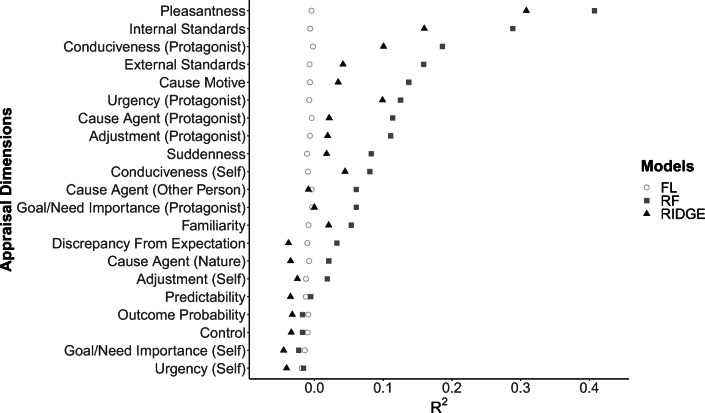
*R*^2^ of the featureless learner (FL), the random forest (RF), and the ridge regression (RIDGE) for the 21 appraisal dimensions averaged over the 20 × 5 cross-validation folds. Appraisal dimensions are sorted by their overall performance

Figure 1 shows the predictive performance of the two machine learning models (RF and RIDGE) and the baseline model (FL) for the 21 assessed appraisal dimensions sorted by the maximum averaged *R*^2^. The featureless baseline model, predicting the mean of the respective appraisal, naturally reached an *R*^2^ of around 0 for all dimensions. The tree-based RF model yielded the best performance for all 21 appraisal dimensions, while the RIDGE performed consistently worse than the RF across all appraisal dimensions. Consequently, the RF was considered the superior model in this context and was used for all further analysis. The RF performance varied strongly between the appraisal dimensions, ranging from −.016 to .407, with *pleasantness* (*R*^2^ = .407) and *internal standards* (*R*^2^ = .289) yielding the highest performance, and *predictability*, *outcome probability*, *control*, *goal/need importance (self)*, and *urgency (self)* the worst performance, with a negative *R*^2^. To rule out the possibility that the differences in the performance achieved were simply due to the different sample sizes between the appraisal dimensions, we calculated a Pearson correlation between the maximum attained *R*^2^ and the sample sizes used for each model. No significant relation was detected (*r*(19) = −.077, *p* = .739).

**Fig. 2:**
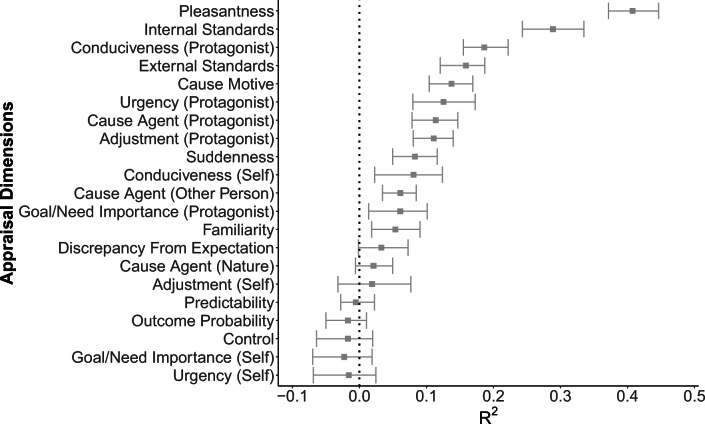
*R*^2^ of the random forest (RF) for the 21 appraisal dimensions, with error bars indicating the 15% and the 85% quantiles of the *R*^2^ attained within the 20 × 5 cross-validation folds. Appraisal dimensions are sorted by their overall performance

The inspection of the performance variation within the folds of the RF model (Fig. 2) showed that in addition to the five dimensions yielding an overall negative *R*^2^, *discrepancy from expectation* (*R*^2^ = .033, 15% quantile = −.002), *cause agent* (*nature*; *R*^2^ = .021, 15% quantile = −.006), and *adjustment* (*self*; *R*^2^ = .019, 15% quantile = −.032) also yielded a negative performance in at least 15% of the folds. Consequently, we considered these dimensions as not robustly predictable and excluded them from further analysis as well.

**Fig. 3:**
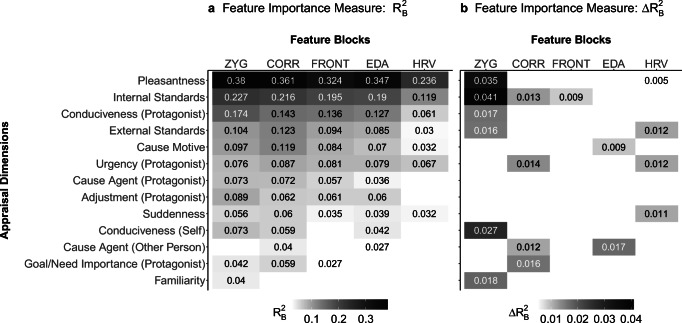
Blocked importance measures ($a:{R}_B^2\ \mathrm{and}\ \mathrm{b}:\Delta  {R}_B^2$) of the five variable blocks (zygomaticus, corrugator, frontalis, EDA, and HRV) for the 13 appraisal dimensions that robustly yielded a positive overall *R*^2^. All importance measures with more than 15% negative or zero values over the 100 iterations are omitted. Appraisal dimensions are sorted by their overall performance

Figures 3 shows the blocked importance measures of the different physiological channels for the appraisal dimensions for which a sufficient overall *R*^2^ was attained. For the first importance measure, ${R}_B^2$, the zygomaticus and corrugator channels overall seemed to contribute similarly to the prediction (*M*_zyg_ = .110, *M*_corr_ = .108). Frontalis, EDA, and HRV performed worse, with HRV having the smallest overall importance (*M*_front_ = .084, *M*_EDA_ = .085, *M*_HRV_ = .044). In 7 out of 13 appraisal dimensions, the zygomaticus channel showed the highest importance value, only yielding no importance for *cause agent (other person).* The corrugator channel yielded the highest importance for the other six appraisals but did not explain any variance for the *familiarity* appraisal. The frontalis channel did not attain a robust positive ${R}_B^2$ for the *conduciveness (self),* the *cause agent (other person),* or the *familiarity* appraisal, while the EDA channel yielded no robust importance for *goal/need importance (protagonist)* or *familiarity*. The HRV channel robustly explained variance for only 7 of the 13 dimensions, contributing nothing to the prediction of *cause agent (protagonist)*, *adjustment (protagonist)*, *conduciveness (self), cause agent (other person)*, *goal/need importance (protagonist)*, and *familiarity*. Naturally, with the decrease in overall *R*^2^, the ${R}_B^2$ attained decreased as well.

**Fig. 4:**
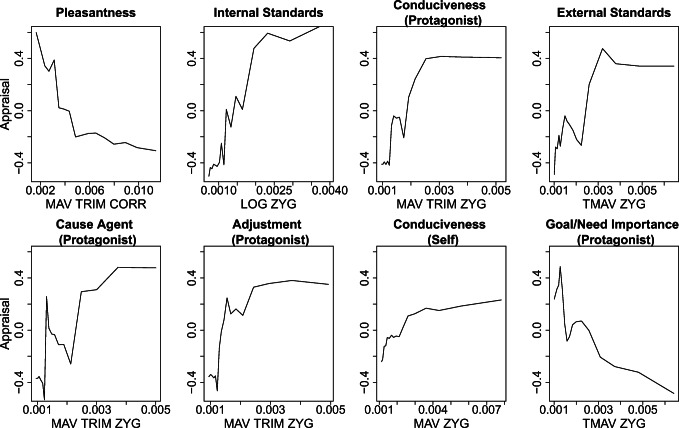
ALE plots for the seven appraisal dimensions for which a feature with robust positive importance was detected. MAV: mean absolute amplitude. MAV TRIM: 20% trimmed mean absolute amplitude. TMAV: mean absolute value attenuated with a moving-window-20%-trimmed-mean filter. LOG: *e* to the power of the mean logarithm of the absolute signal

In the second importance analysis, the $\Delta  {R}_B^2$that represents the uniquely explained variance of the variable block and its interactions, the zygomaticus channel, reached the highest importance across appraisals compared to the other physiological channels (*M*_zyg_ = .012, *M*_corr_ = .004, *M*_front_ = .001, *M*_EDA_ = .002, *M*_HRV_ = .003). The zygomaticus uniquely explained variance for the appraisals *pleasantness*, *internal standards*, *conduciveness (protagonist)*, *external standards*, *conduciveness (self)*, and *familiarity*, while the corrugator channel explained incremental variance for the *internal standards*, *urgency (protagonist)*, *cause agent (other person)*, and *goal/need importance (protagonist)* appraisal. The frontalis channel only reached robust positive importance for the *internal standards* dimension and the EDA channel for *cause motive* and *cause agent (other person)*. Even though the HRV block seemed to have a rather low overall contribution (${R}_B^2$) compared to the other physiological channels, it actually explained variance beyond the other blocks for four appraisals including *pleasantness*, *external standards*, *urgency (protagonist)*, and *suddenness*.

For 5 of the 13 dimensions (i.e., *cause motive*, *urgency [protagonist]*, *suddenness*, *cause agent [other person]*, and *familiarity*), no interpretable feature with robust positive importance could be detected.^6^ Hence, these dimensions were excluded from the ALE plots. For the remaining eight appraisal dimensions, seven zygomaticus amplitude features and one corrugator amplitude feature were selected. All features showed a positive feature importance and hence were able to explain variance in the respective appraisal (*M* = .044, range = .017–.084). ALE plots for the selected features are presented in Fig. 4. *Internal standards*, *conduciveness (protagonist; self)*, *external standards*, *cause agent (protagonist)*, and *adjustment (protagonist)* all showed a tendency towards a positive relationship with the zygomaticus amplitude (i.e., higher ratings of the respective appraisal were related with a higher zygomaticus amplitude). The appraisal *goal/need importance (protagonist),* on the other hand, showed a negative relation with the feature indicating zygomaticus amplitude height. Lastly, the *pleasantness* appraisal showed a negative relation with the corrugator amplitude. For all ALE plots, the type of link can be described as mostly nonlinear.

## Discussion

The present study aimed at exploring how different physiological channels relate to the appraisal dimensions of the CPM (Scherer, 2009) by investigating whether the dimension can be predicted using features extracted from the respective physiological signals. The appraisals were assessed by questionnaire after presenting subjects different emotional video sequences during which the activation of different facial muscles, EDA, and HRV were collected. We compared two different machine learning models—linear and a tree-based—to a baseline model, evaluating which type of model was most appropriate to represent the structure of the data. Moreover, we analyzed the relevance of each physiological channel by constructing two different blocked importance measures. Finally, we took a further step towards making the machine learning models interpretable by looking at ALE plots that depict the relation between an appraisal and a single physiological feature.

The benchmark comparing the predictive performance of the RF and the RIDGE model showed that for 8 out of 21 appraisal dimensions, no robust *R*^2^ was attained. Hence, it can be concluded that the dimensions *discrepancy from expectation*, *cause agent (nature)*, *adjustment (self)*, *predictability*, *urgency (self)*, *outcome probability*, *control*, and *goal/need importance (self)* were physiologically related neither to the activity of the zygomaticus, the corrugator, or the frontalis, nor to EDA or HRV. The theoretical predictions made by the CPM (Scherer, 2009) are to some degree incongruent with these results, as it was theoretically assumed that the *control* appraisal would be related to the activity of different facial muscles such as zygomaticus and corrugator and the *predictability* appraisal to all five assessed channels. We were not able to empirically substantiate these relations in the setting of the present study, where emotions were induced by watching videos. Further, it was noticeable that the *adjustment*, *urgency*, and *goal/need importance* dimensions were predictable, reaching a substantially higher *R*^2^ than the baseline model when appraised from the perspective of the video protagonist. This suggests that the appraisals might be related to the assessed physiological channels, but that in the passive viewing of a video sequence, the appraisal attribution to the protagonist could be more decisive. This would mean that for the affective evaluation of a passively experienced event, it is more important whether one feels that the protagonist of the event can adjust to the consequences, has to react urgently, or is influenced strongly by the events, rather than the appraisal of those dimensions from one’s own perspective. The fact that we were able to predict from the physiological features whether an event was caused by the protagonist or by a different person in the video plot (*cause agent [protagonist]* and *cause agent [other person]* appraisals), but not if the event was caused by natural forces or chance (*cause agent [nature]* appraisal), could mean that the three items (intended to measure a single appraisal or construct) actually constitute separate appraisals—an assumption that is also supported by the insufficient correlations of the items. Alternatively, the appraisal outcome, indicating that an event was caused by nature rather than by a person, might affect different physiological responses that were not considered in the present study.

For the 13 dimensions for which a robust positive *R*^2^ was attained, the RF consistently performed better than the RIDGE. This comparison clearly shows that the relations between the physiological features and the appraisal dimensions cannot be sufficiently represented by a linear model, and are probably nonlinear. This assumption is also supported by the single-feature ALE plots, which showed nonlinear links between appraisal and physiology. Evidence for the nonlinear relationship between physiological features and the valence and arousal evaluation of an event has been demonstrated by Russo, Vempala, and Sandstrom (2013). The authors showed that both dimensions can be predicted with a cross-validated *R*^2^ of 62.4% (valence) or 82.8% (arousal) from physiological features extracted from EDA, HRV, facial EMG, and the respiration rate of a person when using nonlinear neuronal networks. The predictability decreased, however, when a simpler linear model was applied (valence: *R*^2^ = 53.3%; arousal: *R*^2^ = 59.3%). Meuleman et al. (2019), who predicted ratings of hypothetical physiological and expression responses using different appraisal factors, showed that the performance of their models increased to some extent when nonlinearity was added. Hence, a linear model does not seem to provide sufficient complexity to fully display the link between appraisal and physiology and expression. The use of linear models for better interpretability and the linear phrasing of relations derived from theory or empirical studies (e.g., Scherer, 2009) therefore probably constitutes a simplification or could even be misleading.

The out-of-sample *R*^2^ of those dimensions that were robustly predictable varied strongly, ranging from *R*^2^ = .054 for *familiarity* to *R*^2^ = .407 for *pleasantness.* Especially for the dimensions in the lower end of this range, the assessed five physiological measures are probably not sufficient to fully explain their variance. It is likely that those appraisals affect further aspects of physiology that are consequently needed to fully predict them. The reliability of our items is unknown, but our single item measures clearly limit the maximally attainable *R*^2^. Moreover, based on the already mentioned debate on how well automatically processed appraisals can actually be assessed via self-report (Davidson, 1992; Scherer, 1993a, 2005), the measurement by questionnaire might more generally be a cause for increased measurement error in the appraisal data. We nonetheless tried to assess the appraisal process in a less retrospect way compared to the original GAQ (Geneva Emotion Research Group, 2002) by asking participants to rate the appraisal dimensions immediately after the emotional video was viewed in a controlled laboratory setting, hoping to minimize potential measurement error and retrospective biases as far as possible. Due to artifacts and noise, which cannot be fully prevented, measurement error was of course also present in our physiological features to some extent. Considering these assumptions, the performance achieved seems reasonable.

The first blocked importance measure, the ${R}_B^2$, that was implemented to assess how much variance the variables of each channel and their interactions can explain within the 13 appraisals with a sufficient overall *R*^2^, showed that the zygomaticus and corrugator channels contributed similarly to the appraisal prediction and overall seemed to be most important. On average, the frontalis and EDA channels explained less variance than the zygomaticus and corrugator, while the HRV seemed to be the least relevant channel. For the channels that yielded robust positive importance, it can be assumed that a relation between the respective appraisal and the physiological channel exists. Some of these links have already been made by theoretical or empirical work, while others are somewhat contradictory to previous findings. Scherer’s (2009) theoretical assumptions for *pleasantness, suddenness, familiarity, conduciveness*, and *goal/need importance* entail all physiological channels, predicting modifications in facial expressions and skin conductance, as well as cardiovascular changes. These predictions are only partially in line with our findings. All five channels yielded robust positive importance for the *pleasantness,* the *conduciveness (protagonist)*, and the *suddenness* appraisal; hence all channels were connected to these three appraisals. For *goal need/importance (protagonist)* though, variance was robustly explained by only the three EMG channels. A relation between the appraisal and EDA or HRV was consequently not confirmed within the present context. In addition, *familiarity* seemed to be related to only the zygomaticus channel in our study. Previous empirical research on the physiological changes connected to the *pleasantness* appraisal also demonstrated relations to zygomaticus (Aue & Scherer, 2008; Lanctôt & Hess, 2007; Scherer et al., 2019), corrugator (Delplanque et al., 2009; Lanctôt & Hess, 2007; Scherer et al., 2019), and frontalis activity (Aue & Scherer, 2008; Delplanque et al., 2009), as well as to changes in EDA (van Reekum et al., 2004) and HRV (Delplanque et al., 2009). Van Reekum et al. (2004), on the other hand, were not able to find any effect of *pleasantness* on either frontalis activity or HRV. Similarly, Scherer et al. (2019) found no effect on the occurrence of facial action units that are connected to the frontalis muscle. Van Reekum et al. (2004) even cast doubt on whether *pleasantness* is at all relevant in affect-related physiology and whether the dimension influences the ANS. Our results, though, demonstrate that the evaluation of the intrinsic pleasantness of an event is related to changes in both facial EMG and HRV. A more plausible explanation, which is also recognized by the authors, is that the experimental induction of an appraisal by using games or other stimuli is not always effective—this could also be the problem with the study by Scherer et al. (2019) that used fictitious scenarios that participants were asked to imagine in order to induce appraisal outcomes without a manipulation check. Another problem could be that both studies use linear multivariate analysis of variance/analysis of variance (MANOVA/ANOVA) models to analyze these relations—our results though clearly demonstrated that the link between pleasantness and physiological features is substantially better represented by a nonlinear model. For the *conduciveness* appraisal, the impact on corrugator activity (Aue et al., 2007; Aue & Scherer, 2008; Gentsch et al., 2013; Lanctôt & Hess, 2007), zygomaticus activity (Aue et al., 2007; Aue & Scherer, 2008; Lanctôt & Hess, 2007), EDA (Aue & Scherer, 2008; van Reekum et al., 2004), and HRV (van Reekum et al., 2004) has also been demonstrated in several empirical studies. Van Reekum et al. (2004), who also studied the impact of conduciveness on the frontalis muscle, were again not able to determine a significant effect. Even though this finding could also be explained by the already mentioned potential weakness of their design and statistical analysis, as well as by their very small sample size (*n* = 33), it is worth mentioning that the frontalis block in our study also did not explain any variance for the *conduciveness (self)* dimension that was evaluated from the participants’ own perspective, but showed relatively high importance when evaluated from the perspective of the video protagonist—the same was true for the HRV block. Lastly, the link found between the *goal/need importance (protagonist)* appraisal and the zygomaticus and corrugator activity was also confirmed in an empirical study by Aue et al. (2007). Kreibig et al. (2012) reported a medium effect of EDA on *goal/need importance*, which we however could not replicate in our study. For the remaining seven appraisal dimensions, no studies have been conducted to our knowledge. Even though the CPM by Scherer (2009) additionally makes predictions for the *external* and *internal standards* dimensions, the physiological channels analyzed in the present study are not considered as potential outputs. Therefore, we were able to demonstrate here for the first time that the dimensions *internal* and *external standards*, *cause motive*, and *urgency (protagonist)* are also related to changes in facial EMG, EDA, and HRV, and that *cause agent (protagonist)* and *adjustment (protagonist)* are related to facial EMG and HRV. Lastly, we were able to demonstrate that the *cause agent (other person)* appraisal is linked to both corrugator activity and HRV.

With the $\Delta  {R}_B^2$ blocked importance measure, we additionally analyzed how much incremental variance a block could explain beyond the other considered blocks. This analysis adds to the question of whether a dimension has a unique contribution to the prediction of an appraisal dimension, rather than whether the dimension is related to it at all. Therefore, the results are less relevant for the basic research on the physiology of appraisals, but can be used when the most economical modeling of an appraisal physiology link is the goal. The importance measure shows that for each dimension, between one and five channels do not explain incremental variance, which means that the respective channel can be compensated by the other four channels in the model and that excluding the channel from the complete model would not lead to a loss in performance. For *cause agent (protagonist)* and *adjustment (protagonist)*, for example, the variance explained by each of the five physiological blocks could also be explained by the other four channels in the model. Moreover, robust positive channel importance was attained for only 17 of the 65 measures (5 channels × 13 appraisals), which means that in only 17 cases was a channel able to explain variance beyond the other predictors in the appraisal model. This shows that the channels must be correlated to some degree. For 8 of the 13 dimensions, either the zygomaticus or the corrugator block could be removed if all other dimensions were considered, as in these dimensions neither of the two physiological channels yielded robust positive importance. The zygomaticus channel seems to hold a higher share of incremental variance overall, even though both channels, zygomaticus and corrugator, were able to explain a comparable amount of variance in the appraisals in the first importance analysis. Moreover, the frontalis dimension, which also achieved an overall substantial ${R}_B^2$ (*M*_front_ = .084), could actually be removed for all appraisals except *internal standards* without a loss in performance if the other four blocks were included in the model. Similarly, the EDA block could be excluded for all but two considered dimensions. Interestingly, although the HRV block explained less variance (${R}_B^2$) compared to the other physiological signals (M_HRV_ = .044), it actually uniquely explained variance for four dimensions and should therefore not be excluded when modeling the respective appraisals. For the EMG measures, a correlation between two blocks, which leads to shared variance and hence to their interchangeability, could also be caused by crosstalk between facial muscles and does not necessarily imply a true relation—especially for the frontalis and corrugator muscles that are in close proximity to each other, this has to be considered.

In our last analysis, we specifically looked at the type and direction of the relation between each appraisal and the most important amplitude or HRV feature of the respective dimension. The complexity of machine learning models that can account for high-order interactions and nonlinearity is one of the main benefits of these models, but also constitutes an obvious downside—their interpretability. ALE plots are one approach for increasing interpretability by visualizing the influence of a single feature on the prediction of a model. For eight appraisal dimensions, an interpretable feature with a robust positive importance measure was detected. With the resulting eight ALE plots, we were again able to replicate some findings of previous empirical research. Like Aue and Scherer (2008), we found a negative link between corrugator and pleasantness—a result that is also in line with the theoretical assumptions by Scherer (2009). We further found a positive relation between both *conduciveness* dimensions (*protagonist and self*) and zygomaticus activity, which has also been reported by previous studies (Aue et al., 2007; Aue & Scherer, 2008). The finding that *goal/need importance (protagonist)* is negatively related to the activity of the zygomaticus is partially congruent with the findings of Aue et al. (2007), who reported lower zygomaticus activity related to stimuli of cultural threat used to induce goal relevance. However, the authors also reported increasing zygomaticus activity in response to stimuli depicting biological threat, which contradicts our results. As the sample used in this study was rather small (*n* = 42), and as only linear relations were considered, our results might be more reliable. Nevertheless, it is also possible that the induced goal importance scenarios in the study actually constitute two different appraisal dimensions, producing different results. The remaining ALE plots suggest that zygomaticus activity increased overall if events were rated as more compatible with *internal* and *external standards,* when the protagonist was thought to be able to adjust well to the consequences of the events shown (*adjustment [protagonist]*), and when the protagonist of the video was identified as the cause of events (*cause agent [protagonist]*). The ALE plots showed mostly nonlinear relationships, which again indicates that the use of linear models and the subsequent linear interpretation of the resulting relations might be misleading.

### Limitations

The present study has several limitations. We were able to demonstrate that the majority of participants experienced a rather intense emotional episode during the viewing of the video, which indicates that an appraisal process was triggered. Also, a substantial amount of variance was present in the appraisal ratings both between the videos and between subjects. However, it is possible that the specific selection of videos might not have induced the full range in all appraisal dimensions. Also, the use of passive stimuli such as videos or pictures holds some disadvantages, as they are typically not action-oriented, which also could have led to decreased variance in more action-oriented appraisals and hence to decreased predictability of these appraisals. We therefore urge to validate the present results in more action-oriented and less intense contexts.

Moreover, as we measured each appraisal dimension with a single item, we have to assume rather low reliability of our measurements, which probably affected the *R*^2^ obtained in our study. The original GAQ (Geneva Emotion Research Group, 2002) from which items were selected also provides single items for 9 of the 16 included appraisal dimensions, and not more than three for the other 7 dimensions. This means that the factorial validity, the underlying measurement model of the questionnaire, and its reliability also cannot be evaluated. Moreover, the low inter-item correlations for the *cause agent* dimension, which we assessed with all three items from the original questionnaire, indicate that the three items do not load on the same latent variable and that these items rather represent distinct dimensions. For future research, it would be desirable to develop a new self-report measurement tool for the appraisal process that provides multiple items for each appraisal and allows for an evaluation of measurement quality. Research on appraisal theories of emotions, which still relies heavily on self-reported appraisals, would strongly benefit from such a development. However, as many appraisal dimensions are thought to be processed at least partially in an automated fashion, appraisal critics and appraisal theorists alike question whether the appraisal process can be accessed exhaustively via self-report alone (Davidson, 1992; Scherer, 1993a, 2005). Hence, the general reliance on self-reported data for the assessment of the appraisals probably contributes to measurement error in our data as well. It is an obvious paradox that when trying to find a way to assess the appraisal process (or any other contents of cognition) in a more objective indirect way (e.g., based on measures like EMG or by neuroscientific approaches), research cannot avoid asking participants about their inner states. Even when inducing appraisals in an experimental context, we should somehow verify how an event is actually evaluated. This validity problem is unfortunately not fully solvable with currently available measurement tools and the reliability they provide. Measurement error in the physiological channels due to artifacts, noise, and crosstalk is also not fully avoidable, even with thorough preprocessing. Consequently, the model performance in our study could also be limited by impaired physiological features. Potential crosstalk between EMG regions might have also affected the results of our second importance measure by decreasing the incrementally explained variance of some physiological channels.

Because we were only able to assess the appraisal ratings once by self-report (not continuously), we had to aggregate the continuously assessed physiological measurements on a video level as well. Hence, both measures depict only a summary of appraisal and physiology during the video—the respective loss of information most likely also affected the performance levels obtained. To analyze the relationship between appraisals and physiological responses dynamically, and for the development of a continuous appraisal measurement tool, appraisal dimensions need to be measured continuously. To our knowledge, a continuous measurement of subjective appraisal ratings has not been done before in research on appraisal theories—most likely because such a study would be methodologically complicated. However, some studies have continuously assessed valence and arousal ratings of participants using a joystick-based interface (e.g. Li, Baveye, Chamaret, Dellandréa, & Chen, 2015; Sharma, Castellini, van den Broek, Albu-Schaeffer, & Schwenker, 2019)—an approach that could also be applied in the appraisal context. It has to be assumed, though, that such a continuous rating would decrease the reliability of the appraisal measurement even more. The method would moreover be restricted to measuring only one or two appraisals at a time.

As we modeled the appraisal–physiology link in the reverse direction compared to the theoretically assumed causal process, the models did not include variance explained by interactions between the appraisal dimensions. However, there is evidence from the study of Meuleman et al. (2019) that the predictability of some expression factors derived from semantical emotion ratings increased when interactions between appraisal factors were considered; however, these effects were only present for expression factors that were unrelated to the measures in our study. Nonetheless, it could be possible that omitting appraisal interactions could have decreased the reported effects in the present study.

Lastly, as the majority of recruited participants were students of the Ludwig-Maximilians-Universität München, our sample is from a rather specific selected population with a high level of education—this has to be considered when interpreting the results. A validation of the present results on a more representative sample (as regards education) would be desirable.

### Conclusion

In summary, we were able to investigate the connection of several physiological measures to a broad set of appraisal dimensions by using a data-driven machine learning approach. The results of the present study are based on a substantially higher sample size than most of the discussed research on this topic, and all findings were additionally validated on hold-out data and checked for robustness. We were able to replicate some findings of previous research and added new information for those dimensions that had not yet been investigated. We were able to investigate the appraisal–physiology link for six dimensions (*internal standards*, *external standards*, *cause motive*, *urgency*, *cause agent*, and *adjustment*) that have not yet been empirically (or theoretically) analyzed—probably because these dimensions are difficult to test using the appraisal induction designs typically applied in this field of research. Moreover, our results indicate that the links between physiology and affect-related appraisal are nonlinear and that future studies should refrain from using simple linear models, as the results might be misleading. With these new insights, we hope to extend the knowledge base on the appraisal–physiology relation and facilitate further research on this topic.

By analyzing additional physiological channels and their links to appraisals, future research should be able to increase the predictability of appraisal dimensions even more. Overall, the fact that cognitive categories such as the perceived compatibility of an event with laws and social norms (*external standards* dimensions) can be predicted (at least to some degree) by physiological measures is impressive. The results lend support to cognitive theories of emotions, such as the CPM (Scherer, 2009), that assume that emotions are not simply the subjective perception of a bodily response to a stimulus, and that the cognitive evaluation of our environment is the central element in a multi-modal emotion process.

## Footnotes

1 As several different features need to be extracted from each signal to characterize it sufficiently, the reduction of features to a single outcome variable (that would be needed to model the appraisal–physiology link in the theoretically implied direction) leads to a large loss of information and subsequently to an underestimation of the investigated links.

2 For the full set of assessed variables, see the codebook of our preliminary questionnaire at our OSF repository.

3 For the RF, the *ranger* learner from the *ranger* package (Wright & Ziegler, 2017) was used. Per default, the number of trees to grow (ntree) is set to 500, the number of variables considered in each split (mtry) is set to $\sqrt{p}$ (rounded down, where *p* is the number of features in the model; in our case, $\sqrt{134}$is roughly 11), the tree depth (max.depth) is unlimited, and the minimum node size (min.node.size) is set to 5. For the RIDGE, the *cvglmnet* learner from the *glmnet* package (Simon, Friedman, Hastie, & Tibshirani, 2011) was used. To perform a ridge regression instead of a lasso regression, the alpha parameter was set to 0. Per default, the lambda parameter is tuned in a tenfold cross-validation to find the optimal value for the penalty term.

4 To handle the multilevel structure of the data, we compared blocking the data in training and test sets by subjects to an approach in which the features were subject-mean-centered to remove individual biases. The two methods yielded very similar results, with a high correlation of *r* = .98 in their performance over all 21 appraisal tasks. The blocking method, though, showed slightly lower performance on average (the mean deviation from the residualized model was *R*^2^ = .026). Hence, the blocked method was chosen as the more conservative one in this context.

5 The decision to conduct this additional preprocessing step for the linear model was based on the observation that the model’s performance strongly decreased due to extrapolation when outliers were not excluded. Consequently, we deemed the linear model to be not competitive with the random forest model without addressing its sensitivity to outliers. The performance of the nonlinear random forest model proved to be robust to outlier exclusion.

6 We also report a large table with the importance of all features that yielded robust positive importance for all 13 models in our OSF project.
